# Supplementary material for: Atypical Ductal Hyperplasia and Lobular In Situ Neoplasm: High-Risk Lesions Challenging Breast Cancer Prevention
Source: Cancers (Basel). 2024 Feb 19;16(4):837. doi: 10.3390/cancers16040837 (PMC10886664; doi:10.3390/cancers16040837)
Supplement: Supplementary file 1 [file cancers-16-00837-s001.zip › cancers-2824605-supplementary.pdf]

Table S1. Distribution of patients by age group.

Variable	Level	ADH (N=120)	LIN 1/2 (N=126)	P-value
**Age at VABB, median (min-max)**		52 (31-78)	49 (37-70)	0.056
**Age at VABB, N (%)**	<50	47 (39.2)	67 (53.2)	0.028
	≥50	73 (60.8)	59 (46.8)	
**Age at VABB, N (%)**	<45	24 (20.0)	26 (20.6)	0.025
	45-55	47 (39.2)	68 (54.0)	
	>55	49 (40.8)	32 (25.4)	
